# Pterostilbene Ameliorates Hyperhomocysteinemia‐Induced Cognitive Impairment: Involvement of Mitochondrial Dysfunction, Synaptic Plasticity, and Adult Neurogenesis

**DOI:** 10.1002/alz70859_103244

**Published:** 2025-12-25

**Authors:** Bhaskar Jyoti Dutta

**Affiliations:** ^1^ NIPER Hajipur, Hajipur, Bihar India

## Abstract

**Background:**

Homocysteine (Hcy) is a sulfur‐containing amino acid generated during protein catabolism, and elevated levels of Hcy, known as hyperhomocysteinemia (HHcy), have been implicated in various neurological disorders. Disruptions in Hcy metabolism, along with deficiencies in folate and vitamin B12, can lead to altered methylation and redox imbalances, which in turn affect calcium influx and contribute to the accumulation of amyloid and tau proteins—key factors in the development of cognitive impairment. Pterostilbene (PTE), a natural stilbene compound, has demonstrated potential in mitigating neurological deficits due to its antioxidant and anti‐inflammatory properties. This background sets the stage for exploring pterostilbene’s therapeutic potential in the context of HHcy‐induced cognitive decline.

**Method:**

In this study, we developed a model of hyperhomocysteinemia (HHcy)‐induced cognitive impairment (HHcy‐Cog) by administering L‐methionine, a precursor of homocysteine (Hcy). The experimental groups included: normal control rats, HHcy‐Cog rats receiving 1.7g/kg of L‐methionine orally, and HHcy‐Cog rats treated with PTE at doses of 30 mg/kg or 60 mg/kg orally for 30 days. A battery of behavioral tests, including the Morris water maze and novel object recognition tests, were conducted to evaluate various aspects of memory and cognitive function in the treated animals. Following behavioral testing, the animals were sacrificed, and histopathological studies, immunohistochemistry (IHC), and Western blot analyses were performed on brain tissues to further investigate the molecular and cellular changes associated with cognitive impairment and the potential therapeutic effects of PTE.

**Result:**

PTE effectively reversed HHcy‐induced cognitive impairment by significantly improving spatial and recognition memory, as evidenced by performance in behavioral tests. Additionally, PTE was found to enhance mitochondrial biogenesis through the SIRT1/PGC‐1α/TFAM pathway, promoting mitochondrial health. It also improved synaptic plasticity, as shown by increased expression of synaptophysin and PSD‐95, and supported adult neurogenesis, evidenced by upregulation of doublecortin expression in hippocampus.

**Conclusion:**

PTE effectively alleviates HHcy‐induced cognitive impairment by enhancing memory function, mitochondrial biogenesis, synaptic plasticity, and adult neurogenesis. These results highlight PTE's potential as a therapeutic agent for combating cognitive decline in HHcy‐related conditions.

